# Reflex Epilepsy

**DOI:** 10.14336/AD.2021.0216

**Published:** 2021-07-01

**Authors:** Samrina Hanif, Shane T Musick

**Affiliations:** ^1^Department of Neurology, Marshall University, Joan C. Edwards School of Medicine, Huntington, WV 25701, USA.; ^2^Department of Neurosurgery, Marshall University, Joan C. Edwards School of Medicine, Huntington, WV 25701, USA.

**Keywords:** Epilepsy, Reflex epilepsy, reflex seizures, reflex

## Abstract

Reflex seizures (RS) are epileptic events that are objectively and consistently elicited in response to a specific afferent stimulus or by an activity of the patient. The specific stimulus can be a variety of heterogenous intrinsic or extrinsic factors, ranging from the simple to the complex, such as flashing lights or reading a book. These seizures can take a variety of forms, comprising either general or focal onset, with or without secondary generalization. Reflex epilepsies (RE) are classified as a specific syndrome in which all epileptic seizures are precipitated by sensory stimuli. The few designated RE include idiopathic photosensitive occipital lobe epilepsy, other visual sensitive epilepsies, primary reading epilepsy, and startle epilepsy. RS that occurs within other focal or generalized epilepsy syndromes that are associated with distinct spontaneous seizures are classified by the overarching seizure type. Most patients experience spontaneous seizures along with their provoked events. RS originate from stimulation of functional anatomic networks normally functioning for physiological activities, that overlap or coincide with regions of cortical hyperexcitability. Generalized RS typically occur within the setting of IGEs and should be considered as focal seizures with quick secondary generalization via cortico-cortical or cortico-reticular pathways. In aggregate, activation of a critical neuronal mass, supported and sustained by cortico-subcortical and thalamocortical pathways eventually result in a seizure. Treatment includes antiseizure medication, commonly valproate or levetiracetam, along with lifestyle modifications, and when amenable, surgical intervention. High clinical suspicion and careful history taking must be employed in all epilepsy patients to identify reflex triggers.

Reflex seizures (RS) are epileptic events that are objectively and consistently elicited in response to a specific afferent stimulus or by an activity of the patient [1]. The specific stimulus can be a variety of heterogenous intrinsic or extrinsic factors, ranging from the simple to the complex, such as flashing lights or reading a book. These seizures can take a variety of forms, comprising either general or focal, with or without secondary generalization. Reflex epilepsies (RE) are classified as a specific syndrome in which all epileptic seizures are precipitated by sensory stimuli [1-3]. The few designated RE include idiopathic photosensitive occipital lobe epilepsy, other visual sensitive epilepsies, primary reading epilepsy, and startle epilepsy [2, 3]. RS that occurs within other focal or generalized epilepsy syndromes that are associated with distinct spontaneous seizures are classified by the overarching seizure type. Consistently, this clinical phenomenon is common, with most patients with RS also suffering from spontaneous seizures. RS have an overall prevalence rate of 4%-7% in all epilepsy patients, and upwards of 21% in idiopathic generalized epilepsies (IGE) [4].

Epileptic seizures that were clearly precipitated by a specific perceptual stimulus was first appreciated in the 2^nd^ century AD [2]. Thereafter, the first detailed description of reflex events came from Marshall Hall in 1850, initially noting photosensitive epilepsy [4]. The International League against Epilepsy (ILAE) classification system first described the induction of epileptic events closely associated with specific stimuli in 1989 [2, 4]. Subsequently, the 2001 ILAE classification was the first to elaborate the definitions of RE and RS [1]. The newest 2017 ILAE classification makes no specific mention of RS [5, 6]. However, the instruction manual accompanying the new classifications do mention RE and leave room for its continued promulgation [7]. Therefore, under the current ILAE classification, RS receive the same emphasis as spontaneous seizures in an epilepsy diagnosis [8].

The specific factors that cause RS are myriad, and include visual stimuli as the most common, comprising 75%-80% of all RS, along with thinking, reading, music, eating, orgasm, startle, and more [4]. Importantly, seizures induced by drug withdrawal, fever, extreme emotional distress, sleep loss, et cetera are not considered RS, but are reactive [9]. Furthermore, there is a crucial distinction between a precipitating stimulus and a facilitating one, as only the former can be considered a RS; whereas an increase in seizure intensity in response to intermittent photic stimulation (IPS) in certain IGE patients is a facilitating stimulus and thus not a RS [2]. In aggregate, RS originate from stimulation of functional anatomic networks normally functioning for typical physiological activities, that overlap or coincide with regions of cortical hyperexcitability [4, 9]. Previous delineations have dichotomized RE into either focal or generalized [3], but this has been pointed out as a flawed classification system in light of more evidence [9, 10] Therefore, albeit somewhat arbitrary, RE and RS can be designated as occurring in response to either intrinsic or extrinsic stimuli. It must be appreciated that many specific RS triggers occur due to a complex combination of both modalities, however, for ease of classification only the demarcation between intrinsic and extrinsic will be considered, as put forth by Panayiotopoulos [2]. Prior to examining those RS classifications, a brief overview of IGEs and isolated REs will be given.

## Idiopathic Generalized Epilepsy

IGEs overall comprise 25% of all epilepsy syndromes [11]. Based upon recent ILAE classifications, epilepsy syndromes are broken into etiological groups, namely genetic, infectious, structural, metabolic, immune, and unknown [6]. By definition, patients with IGE are devoid of structural brain lesions, and lack signs and symptoms interictally. However, on electroencephalography (EEG) they demonstrate a combination of generalized spike and wave discharges occurring at over 2.5 Hz [11]. The four specific IGEs are: childhood absence epilepsy (CAE), juvenile absence epilepsy (JAE), juvenile myoclonic epilepsy (JME), and generalized tonic-clonic seizures (GTCS) alone [12].

CAE occurs in childhood, is twice as common in females, and has peak onset between 4-7 years of age [13]. It is characterized by brief unprovoked absence seizures, with abrupt onset and cessation, and impairment of consciousness with no post-ictal symptoms [12]. These spells can occur hundreds of times per day. It can be induced by hyperventilation, and physical examination is otherwise normal. JAE peak age of onset is between 9 and 13 and occurs evenly in males and females [13]. In JAE, predominant seizure type is absence, which typically occurs many times per day, but much less frequently than in CAE. A minority of these patients also experience spontaneous GTCS [12]. JME is the most common IGE, which peaks during adolescence between 12-18 years of age [13]. Seizure semiology typically consists of myoclonic jerks of any part of the body, most commonly in the morning. Two-thirds of patients also experience spontaneous GTCS [12]. Rarely do they have absence seizures. Lastly, patients with GTCS alone experience exclusively GTCS. Age of onset is variable, with most occurrences developing between mid-teens to mid-thirties [12, 13]. The three first-line antiseizure medications (ASMs) for the IGEs are valproic acid, lamotrigine, and ethosuximide. Ethosuximide is used primarily to treat CAE, whereas valproic acid monotherapy can achieve seizure freedom in up to 75% of patients with other IGEs [12].

This overarching class of epilepsy is crucial in the consideration of RS, as upwards of 21% of patients with IGEs will experience concurrent RS [4]. Generalized RS in fact typically occur within the setting of IGEs, and therefore should be considered as focal RS with rapid secondary generalization due to focal or multifocal hyperexcitable cortical areas; this allows for generalized spread via cortico-cortical or cortico-reticular pathways [10]. In other, rare cases, isolated or (nearly) purely focal REs can occur, consisting of idiopathic photosensitive occipital lobe epilepsy, primary reading epilepsy, and startle epilepsy. In idiopathic photosensitive occipital lobe epilepsy, all seizures are provoked by photic stimuli, with a benign interictal EEG and physical examination [14]. However, it has become increasingly recognized that some patients will experience rare myoclonic or GTCS as well, especially with prolonged stimulation, indicating this clinical entity as a continuum between focal and generalized events [14, 15]. That sentiment holds true for reading and startle RE, which are considered in detail below.

## Extrinsic Reflex Epilepsy

Extrinsic RE refers to a RS induced by a number of specific stimuli that originate extrinsically and impinge on various sensory perceptual systems. Extrinsic stimuli are those sensory stimuli that originate from the environment. This includes differing forms of photosensitive epilepsy, as well as auditory, olfactory, gustatory, and exteroceptive inputs.

### Photosensitive epilepsy

Photosensitive epilepsy is a nonspecific term comprising the heterogenous group of epilepsies in which RS are elicited by photic stimulation [2]. Television, video games, and light flickering, especially in the deep red band wavelength (680-700 nm) are the most common specific triggers [3, 9]. The likelihood of seizure induction increases with greater luminance of the light source, prolonged light exposure, high-contrast stimuli, binocular stimulation, a flash stimulus of between 15-25 per second, and distinct geometric patterns that are idiosyncratic per patient [4, 16, 17]. It is by far the most common form of RS, with a prevalence of between 2%-10% of all patients with epilepsy, with higher estimates stemming from considering younger patients (7 - to 19-years old) [18, 19], and it is twice as likely in females [9].

Photosensitivity is the distinguishing feature of photosensitive occipital lobe epilepsy, a RE syndrome, and Jeavons syndrome, a reflex syndrome as part of an IGE. Moreover, several IGEs possess photosensitivity as a common reflex trait. For instance, differing estimates point to an incidence of photosensitivity between 30%-90% in JME [20]. CAE is another IGE with frequently reported photosensitivity, ranging between 18%-40% [9, 21]. Further syndromes commonly associated with photosensitivity include childhood epilepsy with occipital paroxysms, Dravet syndrome in up to 70% of patients, Unverricht-Lundborg disease in upwards of 90% of patients, and progressive myoclonic epilepsies (PME), including neuronal ceroid lipofuscinoses with CLN6 mutations, and Lafora disease [2, 4].

Photosensitivity is characterized by a photo-paroxysmal response (PPR) on EEG during IPS. Waltz and colleagues proposed an EEG classification system for photosensitive patients, demarcating between 4 classes: class 1 - occipital spikes; class 2 - local parieto-occipital spikes and biphasic slow waves; class - 3 parieto-occipital spikes and biphasic slow waves spreading to frontal regions; class 4 - generalized spikes or polyspikes and waves [4, 22]. A generalized PPR that consists of multiple spikes or spike-and-wave discharges at a range of flicker frequencies, also termed a photoconvulsive response (PCR), which corresponds to a class 4 response by Waltz et al., is considered to be clinically important [9, 22, 23]. Importantly, resting EEG is typically normal in idiopathic photosensitive epilepsies, with 20%-30% of cases showing paroxysms after brief eye closure, which is akin to IPS [4]. Furthermore, the PPR trait follows an autosomal dominant inheritance pattern with reduced penetrance, with putative loci localizing to chromosomes 6, 7, 13, and 16 [4, 24]. Additionally, the CHD2 gene, located on chromosome 15, has been heavily implicated in photosensitive epilepsy [25].

The clinical manifestations of photosensitive epilepsy as a RS, and the PPR, is highly variable contingent on the presence of underlying epileptic syndromes, as well as idiosyncratic differences in response severity. Overall, 42% of patients with the presence of a PPR along with clinical seizures have pure photosensitive epilepsy devoid of spontaneous seizures, while 40% have both photosensitive and spontaneous seizures, and 18% have spontaneous seizures only, even with a PPR on EEG [2, 26, 27]. The seizure semiology is most often generalized, with myoclonic jerks, GTCS, and absences as the most common manifestation in descending order of frequency [2, 4, 9]. Specific manifestations include visual qualia, such as colorful hallucinations, along with eyelid myoclonus with or without impaired consciousness, myoclonic jerks of the head or body, and absence seizures [2, 9, 10]. More sustained photic exposure can eventually lead to *bona fide* GTCS. Less commonly, occipital focal seizures without secondary generalization can occur, which is an isolated RE syndrome.

The mechanism of photic-induced seizure initiation depends upon excitation of a critical neuronal mass within the occipital cortex, coupled with intrinsic hyperexcitability of the visual cortex to facilitate large-scale neuronal synchronization [4, 9]. To allow for such a generalized response, there are also diffuse or multifocal hyperexcitable pathways, including cortico-cortical, or cortico-subcortical, that aid in epileptic propagation [4, 10]. Diffuse inadequacy of GABAergic inhibition is a proposed mechanism underlying intrinsic hyperexcitability and network synchronization. Consistently, enhanced gamma-band phase synchrony precedes a PPR in photosensitive patients [28], and gamma oscillation frequency correlates with resting concentrations of GABA in the visual cortex [9, 29]. Dopamine inadequacy is an additional neurotransmitter candidate, with findings that apomorphine, a dopamine receptor agonist, could block the PPR in photosensitive patients, but had no impact in non-photosensitive ones [2]. After a local epileptic discharge is elicited in the occipital cortex, specific pathway propagation may include cortico-reticular or cortico-cortical pathways, with involvement of parieto-occipital areas to cause generalized discharge [10]. Congruent with this, a cortico-cortical propagation across the intraparietal sulcus, connecting the occipital to premotor cortex underlies the PPR [30]. In an EEG-fMRI study in patients with JME, Bartolini et al. demonstrated that the putamen can act as a mediator between the visual and motor system during a generalized PPR [31]. Differing pathways likely reflect a combination of frank diffuse or multifocal hyperexcitability, along with distinct pathways in different syndromes.

### Television-Induced Seizures

TV-induced seizures first received major prominence following the infamous Pokémon incident in Japan in 1997, whereby photosensitive seizures reached epidemic proportions following showing of an animated cartoon [32]. TV-induced seizures are the most common specific extrinsic stimulus to induce photosensitive seizures, and are the most common type of photosensitive epilepsy [4]. They are twice as common in females and typically affect children aged 10-12 years-old, with upwards of 10% of afflicted patients harboring a family history of TV-induced seizures [2, 4]. Seizures are more likely with screen flickering, close proximity to the screen (< 1 m), greater light intensity, and darker rooms to impart greater screen contrast [2, 4, 33]. Flicker frequency is the most salient factor in photic-induced seizures. Specifically, lower frequency TV-sets are more likely to induce seizures [2, 4]. For this reason, LCD and plasma-screen TVs are less likely to trigger a seizure due to technological differences from past TV models that prevent the occurrence of flickering [4]. LCD TVs are less likely than plasma screens. It also appears that 3D movies do not trigger seizures more readily than their 2D counterparts in photosensitive patients [34]. Finally, 10% of patients with TV-induced seizures have “compulsive attraction”, whereby they are “drawn like a magnet” to the TV screen before advancing to a GTCS [2, 4].

### Video Game-Induced Seizures

Video game induced seizures were first described in 1981 when a 17-year-old boy developed seizures after playing an arcade game, hence the term “space invader” epilepsy [35]. Thereafter, a multitude of reports were published showing seizure occurrence from a variety of video games [36]. This phenomenon is more common in boys, and typically affects patients between 7-19 years-old. Importantly, video-game induced seizures occur even in response to hand-held LCD display games, and are not limited to TV monitors [2]. Photosensitivity plays the predominant role in seizure induction to video games, and up to 70% of patients with video-game induced seizures display *bona fide* photosensitivity [4]. However, 30% of these patients do not develop a PPR to IPS [37]. In susceptible patients, playing Super Mario World elicited a greater response than did IPS alone [36]. Clearly, more mechanisms are at play than pure photosensitivity, and these include pattern sensitivity, proprioceptive stimuli (movement/praxis), cognitive stimulation (decision making), and emotional excitation (anxiety, frustration, excitement) [2, 4, 36]. Pattern sensitivity is closely related to photosensitivity and seldom occurs in isolation, with most patients displaying a concomitant PPR, along with generalized seizure induction in response to sharply contrasting stripes or lines [2, 9]. Several facilitating factors also play a key role to lower seizure threshold, such as fatigue, prolonged playing, and sleep deprivation [2, 4]. Two thirds of these patients have generalized seizures, with the other third displaying focal occipital seizures.

### Self-Induced Seizures

Self-induction of seizures is a rare and puzzling disorder that occurs nearly exclusively in photosensitive and pattern sensitive individuals [2, 9], albeit there are other reported cases in response to a variety of other stimuli. These patients self-induce seizures via producing optimal epileptogenic conditions of lighting and pattern conditions. The most known example is sunflower syndrome, whereby patients look at a bright light source (typically the sun) and voluntarily wave their abducted fingers in front of their eyes [2]. This activity produces an ideal IPS, along with a degree of pattern sensitivity. Other behaviors involved in self-induction involve repetitive opening and closing of the eyes, specific head movements to make a TV-picture “roll”, quickly changing channels, etc. [2]. Self-induction habits are oft described as a compulsive behavior, with magnetic-like attraction to light sources commonly reported [9].

### Management of photosensitive epilepsy

In patients with pure photosensitive epilepsy, it is recommended they avoid specific triggers as a first line treatment. Specifically, patients with TV-induced seizures should watch from a distance of at least 2 m, only use a remote control to change the channel, watch in a highly illuminated room, utilize a 100 Hz TV or LCD/plasma TV, and limit screen time when somnolent or lethargic [4, 10]. Covering one eye can also be effective, especially when exposed to discotheque stroboscope lights, as monocular stimulation diminishes the seizure propensity. Additionally, wearing a type of blue lens can control the PPR response in most photosensitive patients [10], and wearing polarized glasses during sunny days can also aid in seizure control [4]. Patients with video-game induced seizures should not play when somnolent, lethargic, or sleep deprived, or for excessive periods of time [2]. Additionally, tinted glasses while playing can mitigate the chances of seizure induction. If these prophylactic measures do not provide adequate seizure control, or if the patient has an IGE coupled with photosensitivity, ASM is required. First line medical therapy is typically valproic acid, which can lead to cessation of seizures in the majority of patients [2, 10]. Levetiracetam, brivaracetam, and lamotrigine can also be highly effective and have been demonstrated to diminish the PPR [10]. Lamotrigine treatment can exaggerate jerks [38], and phenytoin treatment has exacerbated seizure occurrence in one pediatric photosensitive patient [39], therefore, these ASMs are not as recommended. Currently, valproic acid, followed by levetiracetam treatment are most recommended. For self-induced seizures, psychiatric therapy is necessitated. Long-term prognosis improves throughout life even when a PPR remains present. Photosensitivity tends to decrease with time and may disappear after the third decade in upwards of 30% of patients [10].

### Musicogenic Epilepsy

Musicogenic epilepsy is an exceedingly rare form of complex RE, with an estimated prevalence of 1:10,000,000 individuals, and a female preponderance [3, 4]. 13% of patients have isolated musicogenic RE, while the majority have both spontaneous and music-induced seizures [10]. There is a wide range of musical and audiogenic stimuli that elicit RS, with variability in the type of music, artists, instrumentation, emotional salience, and timespan of listening, as well as different sounds, such as machinery, church bells, telephones, or simply thinking about a particular song or melody [4]. This highlights the large degree of patient-specific idiosyncrasies at play for eliciting these RS. The seizures are most commonly focal, with or without impaired awareness, arising from the temporal lobes, with rare secondary generalization. While not completely elucidated, PET and EEG studies have demonstrated major involvement of the right hemisphere temporal regions [10]. One fMRI/EEG study showed that emotionally charged music was necessary to provoke a RS, with accompanying activation of right fronto-temporo-occipital networks, whereas neutral music activated only the right primary auditory cortex without seizure provocation [40]. Consistently, it has been hypothesized that a dysregulated hippocampal-prefrontal cortex pathway, mediated through altered dopaminergic signaling, stress, and emotional dysregulation may underlie musicogenic epilepsy [41]. First line treatment is simple avoidance of the known auditory stimulus. If avoidance is either impossible or ineffective, first line ASMs include carbamazepine and lamotrigine, with oxcarbazepine, valproic acid, and levetiracetam representing alternatives or choices for combination therapy [4, 10, 41]. If eligible, patients may undergo temporal lobe resection.

### Reading Epilepsy

Reading epilepsy is a rare form of RE with male preponderance that typically manifests in early adulthood or late adolescence [3]. It typically manifests with myoclonic jerks of orofacial and masticatory muscles, which often causes stiffness and a clicking sensation whilst reading [4]. With unabated stimulation, this can evolve into GTCS. This likely represents a subtype within a larger umbrella of language-induced epilepsy, as neurally related tasks, such as speaking (especially argumentative speeches) and writing, can induce the same seizure semiology. It follows an autosomal dominant inheritance with incomplete penetrance [3, 10]. Interictal EEG is normal in 80% of patients, with 11% displaying spontaneous spike and wave discharges, and another 5% demonstrating temporal lobe paroxysmal discharges [10]. The precise underlying ictogenic mechanism is unclear, but recruitment of a critical mass of language-related areas, with subsequent spread and synchronous discharge is necessary [9]. In general, an increased difficulty or emotional content of the material elicits the RS more readily, which would necessarily involve more activation in a wider network of distributed areas. Several distinct areas have been implicated, however, Brodmann area 6 is a robust candidate; it relates the cognitive activity of grapheme-to-phoneme transition to seizure activity, and it connects with and drives activity of the thalamus, claustrum, and right inferior frontal gyrus during seizure activity [10]. This type of RE has a good prognosis, as the precipitant stimulus can be modified. If necessary, valproic acid or clonazepam can achieve adequate seizure control [4, 10].

### Eating-induced seizures

Eating-induced RS are rare, with an estimated prevalence of 1:1,000 - 1:2,000 among patients with epilepsy [42]. These seizures almost solely occur alongside spontaneous seizures. Specific RS triggers are stereotyped for each patient and may differ between patients. However, Koul and colleagues showed that the precipitating factors were related to mastication and the act of food consumption in 98% of patients in their cohort [43]. Other factors also play key roles in seizure generation, including emotional and autonomic components of eating, olfactory stimulation, extent of gastric distention, and stimulation of the mouth and/or pharynx [4, 10]. In Sri Lanka, there has been a high incidence of eating epilepsy with familial clustering, and the specific trigger has been linked to eating bulky meals with high carbohydrate composition [44]. Seizures are typically focal with or without impaired awareness, and arise within the temporolimbic or extratemporal perisylvian regions, with potential for secondary generalization [10]. Importantly, they do not recur within the same meal [4, 10]. Management involves avoidance of the specific stereotyped eating pattern that elicits the RS per patient, such as modifying the sensory characteristics of the food (drinking through a straw, eating smaller bites, etc), or becoming less engorged [10]. Clobazam can be an effective medication when taken before meals. If ineffective, patients can be worked up for possible surgical intervention.

### Hot water or bathing epilepsy

RS induced by hot water is the second most common form of RE overall, and the most common form of RS in southern India [3]. Certain rituals that occur in southern India, whereby hot water (40º-50º C) is repeatedly poured on top of the head has led to a much higher prevalence in that area [45]. The seizures may originate anytime along the lifespan and have a male predominance [4]. It typically manifests as a focal onset impaired awareness seizure, with rare secondary generalization - they begin *ab initio* with a stunned look and senseless speech, feelings of fear, auditory and visual hallucinations, and complex automatisms [4]. Water temperature and quantity, especially water pouring on the head, are the most important precipitating factors [4, 10]. The pathophysiology may involve damage to the hypothalamic thermoregulatory centers, and involve a degree of hyperthermic kindling [4]. Interictal EEG is most often normal, however, 20%-25% of patients display epileptiform aberrations over the temporal region [3, 4]. Moreover, SPECT studies demonstrated ictal hypermetabolic uptake in the medial temporal structures and the hypothalamus [46]. Management consists in lowering water temperature in baths and showers, and if ineffective, utilizing clobazam 1.5-2 hours prior to taking a bath [4, 10]. Upwards of 1/3 of patients eventually develop spontaneous seizures concurrent with their hot water RE, and in those patients, continuous ASM therapy with carbamazepine, lamotrigine, phenytoin, phenobarbital, oxcarbazepine, valproic acid, or levetiracetam is indicated and can aid in achieving seizure control [4].

### Orgasm-induced seizures

Seizures evoked post-orgasm are exceedingly rare and occur by far more commonly in women. The RS are typically focal onset impaired awareness, and originate from the right hemisphere, with most patients harboring both spontaneous and orgasm-induced seizures with localization-related epilepsy [10, 47]. These RS can occur anywhere from minutes to hours following orgasm. The delayed RS following climax, coupled with no epileptiform activity on EEG following hyperventilation seems to preclude a key role for hyperventilation in seizure initiation [4, 10]. Instead, it is believed that orgasm triggers already sensitized neurons within the network localized throughout various responsible centers of the brain, resulting in seizures [4, 10]. The massive female preponderance also suggests an influence of sex hormones, along with anatomic sexual dimorphism involving the limbic temporal region [10]. Management consists of ASM for the coincident focal seizure disorder, and in some cases, surgical intervention has been effective [10].

### Somatosensory stimulation-induced seizures

RS elicited by somatosensory stimulation are typically due to a specific stimulation, such as skin friction (rubbing epilepsy), tooth brushing (tooth-brushing epilepsy), touching or tapping, pricking, or stimulation of the external ear (auricular epilepsy) [3, 4, 10]. The preponderance of cases arises from a specific hypersensitive cutaneous trigger zone, most often from trigeminal and upper extremity somatosensory regions [3, 10]. The seizures are usually focal onset aware, and begin with a sensory aura, followed by a sensory Jacksonian seizure with tonic motor manifestations, suggesting a supplementary motor area seizure [4, 10]. Secondary generalization occurs infrequently. Cortical lesions of the postcentral gyrus are commonly found in these patients. Tooth-brushing epilepsy patients demonstrate hypoperfusion in the right mesial temporal lobe along with right hippocampal atrophy [3]. Patients with rubbing epilepsy display bilateral spike and wave activities on EEG which instantiates as generalized myoclonic jerks provoked by tapping [48]. The treatment is identical as for focal seizures, with continued ASM utilization.

## Intrinsic Reflex Epilepsy

Intrinsic RE refers to a RS evoked by purely intrinsic processes devoid of predominantly extrinsic sensory triggers. This largely includes stimuli comprising higher brain functions and specific actions or activities performed by the patient. This includes thinking (noogenic) and cognitive function, math (epilepsia arithmetica), startle, praxis, or proprioceptive input.

### Thinking (noogenic) epilepsy

RS induced by non-verbal cognitive activities, aka thinking (noogenic) epilepsy is a rare form of RE that most frequently affects male adolescents [3]. The most common effective triggers include performing calculations, solving a Rubik cube, thinking, making decisions, abstract reasoning, playing chess, playing cards, or drawing complex figures [3, 4]. RS induced by mathematics or spatial tasks are among the most reliable triggers, occurring in 72% of these patients [10]. The particular importance of the spatial component of tasks has been shown by Wilkins et al., which demonstrated that longer and more complex calculations more readily provoked seizures [4, 49]. However, this may also reflect greater and more ubiquitous recruitment of relevant cortical structures, as the tasks eliciting RS are typically higher-order complex cognitive stimuli. Simple calculations recruit only the dominant inferior frontal lobe and angular gyrus, whereas complex calculations recruit bilateral parietal lobes [10].

The seizure manifestation is a GTCS in 96% of patients, typically preceded by myoclonic jerks. Myoclonic jerks alone occur in 76% of patients, and absence seizures coincident with myoclonic jerks in 60% [3, 4]. In upwards of 76% of patients, infrequent spontaneous seizures also occur. In 68% of patients, EEG will demonstrate generalized epileptic discharges. When focal anomalies are present, they most commonly occur over the right frontal or parietal regions [50]. Congruent with this, in one patient with thinking-induced epilepsy, EEG recordings demonstrated bilateral synchronous polyspike and wave discharges, with variable temporoparietal or frontal focal abnormalities seen [49] Accordingly, EEG findings, clinical data, and inheritance pattern implicate this RE as congruent with a primary IGE, most akin to JME or childhood absence epilepsy. Consistently, a PPR is present in 32% of these patients [10]. Minimizing exposure to the stimulus is often challenging, thus valproic acid or other ASMs are typically utilized to achieve seizure control [4, 10].

### Praxis-induced reflex seizures

Praxis-induced RS are essentially a subset of thinking (noogenic) RE with added emphasis toward the planning and subsequent motor component (praxis) of complex tasks. This type of RE was first put forth by Inoue and colleagues, whom stated that these RS are provoked by mulling over complex spatial tasks, making a decision, then responding by using a part of the body [51]. This has been corroborated, with other reports showing that cognitive tasks requiring use of the hands, including written calculation and spatial construction, were more epileptogenic than cognitive activities devoid of any movement component [52]. This includes drawing, playing cards, playing chess or other board games, or using a Rubik’s cube. As with thinking (noogenic) RE, these seizures are typically generalized and often appear as a manifestation of JME [4]. The relevant networks involved in praxis-induced seizure generation is the parietofrontotemporal network, which includes the intraparietal sulcus, the superior parietal sulcus, the inferior parietal lobule, the middle frontal gyrus, the premotor cortex, and the inferior frontal cortex [4]. Clearly, praxis involves more cerebral areas than those involved in purely thinking RE lacking a motor response.

### Proprioception-induced seizures

Proprioception involves continuous top-down prediction errors about limb location, allowing guidance of movements, thus it involves a complex array of extrinsic and intrinsic factors [53]. This type of RS most commonly occurs as a transient phenomenon in patients with nonketotic hyperglycemia, evoked by a change in posture or movement [3, 10]. Seizures are usually focal onset aware and involve the sensorimotor area. Moreover, in patients with sensorimotor or supplementary motor area lesions, proprioceptive stimuli can induce focal seizures [4]. In patients with gait-induced epilepsy, EEG displays maximal electronegativity at the central vertex electrode [54]. In general, these RS begin gradually and initially have sensory Jacksonian manifestations [4]. Nonketotic hyperglycemia patients respond robustly to insulin therapy and seizures do not recur; however, when required or when due to a focal lesion, carbamazepine and clobazam can be effective therapeutic choices.

### Startle-induced seizures

Seizures precipitated by startle are evoked by sudden and unexpected sensory stimuli. This type of RS is rare, with the usual onset in childhood to early adolescence, affecting both sexes equally. The specific stimulus is typically auditory, although in a minority of patients somatosensory and visual stimuli may be a trigger [2]. Sudden noise as opposed to pure sound is the most effective auditory stimulus [3]. The seizures are characteristically focal onset aware and last < 30 seconds, with an initial startle response followed by axial unilateral or bilateral tonic posturing, often with associated autonomic phenomena [3, 10]. These responses frequently result in traumatic falls. The majority of patients are afflicted by static neurological and intellectual handicaps, with infantile hemiplegia predominating [2, 3]. These patents usually have large brain lesions, and the insults typically occur pre- or perinatal, or within the first two years of life. It also frequently occurs in the setting of metabolic diseases and are especially common in the setting of trisomy 21. However, some neurologically normal individuals with startle-induced RS have been reported [55]. Spontaneous seizures concomitantly occur in nearly all of these patients, but they are infrequent and often precede or follow the startle-induced RS [2]. Interictal EEG reflects the underlying structural brain lesion, consisting of diffuse or focal aberrations. Ictal EEG comprises an initial vertex discharge, followed by diffuse relative flattening or low voltage rhythmic activity of 10 Hz [2, 10]. Depth electrodes demonstrate an initial high-amplitude response over the region corresponding to vertex scalp activity, followed by epileptic discharge from the motor or premotor lesioned cortex, then spreading to the ipsilateral mesial frontal and parietal regions, and the contralateral frontal regions [2, 10]. In another report, a combined MEG/video EEG study implicated the supplementary motor area and cingulate gyrus as the genesis of startle-induced RS [56]. Consistently, these seizures clinically resemble supplementary motor area seizures. The underlying network pathophysiology is unknown. However, it may involve a similar network alteration to that of trisomy 21, in which a dysfunction of the nucleus reticularis pontis caudalis causes an exaggerated startle response, which leads to an increase in proprioceptive feedback to an intrinsically hyperexcitable motor cortex, causing seizure [4, 10]. The prognosis for these patients is poor. Adequate seizure control is typically impossible with ASM treatment. Clobazam, lamotrigine, carbamazepine, and levetiracetam may be useful agents [10]. In one study of 4 patients, added lamotrigine treatment to other agents significantly improved seizure control [57]. Certain patients may benefit from surgical intervention, including lesionectomy, callosotomy, or multiple subpial transections [10].

## Animal Models

Several pertinent animal models of different RE have been identified and utilized in research, especially in the quest for efficacious ASMs. The *Papio papio* baboon has emerged as a good model of photosensitive RS derived through natural selection in the wild, as they harbor a natural photosensitivity. Importantly, these baboons are strikingly more photosensitive than other primates from adjacent regions, indicating a strong genetic component [3, 24]. However, no specific genetic studies have been undertaken heretofore to identify the precise genes involved. IPS stimulation at 20-30 Hz induces bilateral myoclonus, preceded by paroxysmal discharges in the fronto-rolandic cortex, with secondary generalization to GTCS [3, 24]. Interestingly, distinct from human photosensitivity, the visual cortex is not the seizure generator. Instead, it plays a permissive role in allowing the motor cortex to discharge paroxysmally and induce seizures [3]. These baboons also display spontaneous seizures not induced by photic stimulation, in analogy with human IGEs.

The epileptic strain of Fayoumi chickens (Fepi) display both photosensitive and audiogenic induced RE. These animals possess an autosomal recessive gene mutation in the synaptic vesicle glycoprotein 2A, which has never been reported in humans [4]. The seizure manifestation consists of stimulus-locked motor symptoms (myoclonus), followed by generalized, self-sustaining convulsions [3, 24]. EEG corroborates the clinical picture, with findings of neurons in the prosencephalon burst-discharging at rest, and neurons in the mesencephalon burst-discharging during seizures [3].

Various mouse models have been identified as models of audiogenic-induced RE. The frings mice carry spontaneous mutations in the MASS1 (monogenic audiogenic seizure-susceptible) gene on chromosome 13. The protein product has high expression levels along the superior and inferior colliculi, facilitating the spread of audiogenic RS [4, 24]. Dilute Brown Agouti coat color mice (DBA/2J) harbor germane mutations on chromosomes 4, 12, and 17, particularly affecting the Asp-1 and Asp-2 genes. In particular, these genes are believed to alter the activity of the Ca^2+^- ATPase channel. This leads to seizures after exposure to a loud mixed-frequency sound (12-16 kHz, 90-120 dB), characterized by initial wild running with subsequent clonic convulsions and a tonic extension [24]. Black Swiss mice possess mutations on chromosome 10 in the JAMS1 (juvenile audiogenic monogenic seizures) gene and have audiogenic-induced RS that are easily induced early on in life and gradually disappear by adulthood. The Lgi1 (Leucine-rich, glioma inactivated 1) knockout and mutant (missense L385R mutation) mouse models lead to autosomal dominant focal epilepsy with audiogenic RS. The homozygous knockout model also presents with spontaneous seizures, whereas the heterozygous mice only display increased susceptibility to sound. This suggests the heterozygous knockout strain can be a model for human autosomal dominant lateral temporal epilepsy with audiogenic RS [24]. The genetically epilepsy-prone rats (GEPRs) consist of a moderately epileptic GEPR-3, and severely epileptic GEPR-9 strain. These rats have alterations of their GABAergic system within the central nucleus of the inferior colliculus, which seems to drive audiogenic seizure activity [24]. However, the alteration is in the form of increased numbers of GABAergic neurons, suggesting that this could represent a compensatory mechanism to combat seizure propagation, or potentially be an epiphenomenon. The Wistar Audiogenic Rats (WARs) model display RS in response to audiogenic stimuli, consisting of wild running, jumping, falling, and finally GTCS. The Krushinsky-Molodkina (KM) rat strain is sensitive to sound, with easily induced RS, characterized by wild running and subsequent GTCS. Genetic Audiogenic Seizure Hamsters developed in Salamanca (GASH-Sal) manifest audiogenic-induced RS. They possess alterations in their KCC2 K^+^/Cl^-^ cotransporter, which causes a dysfunctional GABAergic system to induce seizure susceptibility [24]. Importantly, in certain patients with epilepsy, mutations in the KCC2 gene have been identified [58]. Recently, feline audiogenic RS have been described [59].

In monkeys, the role of proprioceptive afferents of muscular origin in eliciting RS has been demonstrated. After chronic administration of alumina cream to cortical areas representing the hind limb, RS can be provoked by active or passive moment, tapping the muscle, or stretch reflexes. The same has been shown for face cortical regions, with provoked RS induced by chewing or mouth opening [3].

## Current Therapies and Future Directions

The preponderance of patients that experience RS also experience spontaneous events, many within the scope of other epilepsy syndromes. As such, the treatment is the same as for all epilepsies that are not amenable to surgical intervention: broad-spectrum ASMs to increase inhibition and/or attenuate hyperexcitability. The most commonly utilized medications that possess good efficacy include valproic acid, levetiracetam, lamotrigine, and in certain cases, benzodiazepines [9, 10]. In some patients and in certain epilepsy syndromes, lamotrigine, carbamazepine, oxcarbazepine, gabapentin, pregabalin, and tiagabine can exacerbate myoclonic jerks, largely removing these as viable choices for many RS [9]. The seizure-reducing response is similar to other epilepsy syndromes, especially IGEs, and can generally achieve adequate seizure control in up to two-thirds of patients. However, that responsiveness, along with general prognosis in RE, is highly variable due to wide clinical heterogeneity, depending mostly on the specific seizure disorder the RS occur within. Lifestyle modifications, including stimulus avoidance, or the use of special glasses in photosensitive patients is paramount to seizure control. Because the reflex stimulus is highly specific and stereotyped per patient, this can have a tremendous impact on quality of life and seizure reduction. As in all cases of epilepsy that remain recalcitrant to medications and lifestyle changes, epilepsy surgery should be pursued. The most commonly cited RE that often have surgical indications and good responses thereafter include startle-induced RE, musicogenic RE, eating RE, and orgasm-induced RE [10].

RS remain an intriguing field of study to explain the pathophysiology of epilepsy in general. Understanding inhibition of RS may lead to better understanding of spontaneous events. For instance, the stimulus underlying the pathological response and seizure induction can also terminate that same seizure once initiated, and this holds for a variety of stimuli [9]. Already, sensory, motor, or other stimuli is known to interrupt or impede seizure propagation in patients with spontaneous seizures, and specifically certain IGEs. The timing of stimulus vis-à-vis the state of the system is key. Thus, RS and their specific stimuli and temporal connections makes for an ideal model to develop and enhance algorithms for seizure prediction and enable better neuromodulation technology. Other avenues of future research should attempt to investigate the potential continuum between reflex and spontaneous seizures [60, 61]. Related, the idea of RS as variants of the same or similar underlying problems [41]. These could be explored via EEG and fMRI in large groups of patients with the same RS, differing RS, and spontaneous seizures, and explore seizure initiation with brain mapping.

## Conclusions

RS as a whole are seizures that are objectively and consistently demonstrated to be elicited via a specific afferent stimulus or by patient activity. A RE syndrome is one in which all or nearly all RS are precipitated by sensory stimuli or patient activity. RS occurring as a part of focal and generalized epilepsy syndromes with spontaneous seizure occurrence are designated by the overarching seizure type. Moreover, isolated RS can occur in situations that do not require a diagnosis of epilepsy.

RS represent an intriguing clinical phenomenon in which meticulous patient questioning must be undertaken to identify the specific environmental triggers. RS are a common component of various IGEs, and as such, high clinical suspicion and careful history taking must be employed. Even greater clinical suspicion and investigation is required to identify those patients with pure RE or isolated RS. Generalized RS typically occur within the setting of IGEs and should be considered as focal seizures with quick secondary generalization via cortico-cortical or cortico-reticular pathways [10]. These IGE patients with generalized RS possess focal, or multifocal, areas of hyperexcitable cortex that when stimulated induce seizures. Avoiding the specific trigger is paramount for long-term prevention of RS. When behavioral modification is impossible, or when spontaneous seizures cooccur, ASM treatment is required for adequate seizure control. The most commonly utilized drugs for these purposes include valproic acid, levetiracetam, and lamotrigine.
